# Three‐dimensional damage morphologies of thermomechanically deformed sintered nanosilver die attachments for power electronics modules

**DOI:** 10.1111/jmi.12803

**Published:** 2019-05-20

**Authors:** P. AGYAKWA, J. DAI, J. LI, B. MOUAWAD, L. YANG, M. CORFIELD, C.M. JOHNSON

**Affiliations:** ^1^ Department of Electrical & Electronic Engineering University of Nottingham, University Park Nottingham UK; ^2^ Dynex Semiconductor Ltd Doddington Road Lincoln UK

**Keywords:** Correlative microscopy, die attachment, microstructure, power electronics, reliability, sintered nanosilver, thermomechanical fatigue, X‐ray tomography

## Abstract

A time‐lapse study of thermomechanical fatigue damage has been undertaken using three‐dimensional X‐ray computer tomography. Morphologies were extracted from tomography data and integrated with data from microscopy modalities at different resolution levels. This enables contextualization of some of the fine‐scale properties which underpin the large‐scale damage observed via tomography. Lateral views of crack development are presented, which show networks analogous to mud‐cracks. Crack fronts which develop in the most porous regions within the sintered attachment layer travel across the boundary into the copper substrate. The propagation characteristics of these cracks within the substrate are analysed. Evidence is provided of heterogeneous densification within the sintered joint under power cycling, and this is shown to play a major role in driving the initiation and propagation of the cracks. Examination of the texture (differing levels of X‐ray absorption) of virtual cross‐sectional images reveals the origins of the nonuniformity of densification. Finally, cracks within the sintered joint are shown to have a negligible impact on the conduction pathway of the joint due to their aspect ratio and orientation with respect to the assembly.

**Lay Description:**

This paper concerns the use of three‐dimensional (3D) X‐ray tomography, a nondestructive technique, to perform cradle‐to‐grave studies of sintered nanosilver die‐attachments under operation. Sintered nanosilver die‐attachments have been proposed as a more reliable and environmentally friendly alternative to solder alloy joints for emerging power electronics module designs. However, their degradation mechanisms are not as well understood. This same sample‐study is about observing how the fine‐scale structure of a sintered attachment evolves and degrades over time. Using 3D tomography affords otherwise infeasible perspectives, such as virtual cross‐sections in the lateral plane of the attachment. These perspectives provide qualitative information which elucidates the degradation mechanisms. They demonstrate, for example, that the structure of the sintered attachment densifies under operation, and a consequence of this is the formation of shrinkage cracks in the most porous regions, much like mud‐cracks. Other imaging techniques (metallographic etching and scanning electron microscopy) have been used in correlation with 3D renderings of these cracks to analyse their propagation and reveal their relationship both with the internal structure of the sintered attachment itself, and the structure of the substrate to which it is joined. It is shown that the cracks develop within the sintered attachment layer and eventually cross over into the substrate. A comparison of two sintered attachments with contrasting bulk porosities allows the effect of initial bond quality on crack development to be examined.

## Introduction

**Figure 1:**
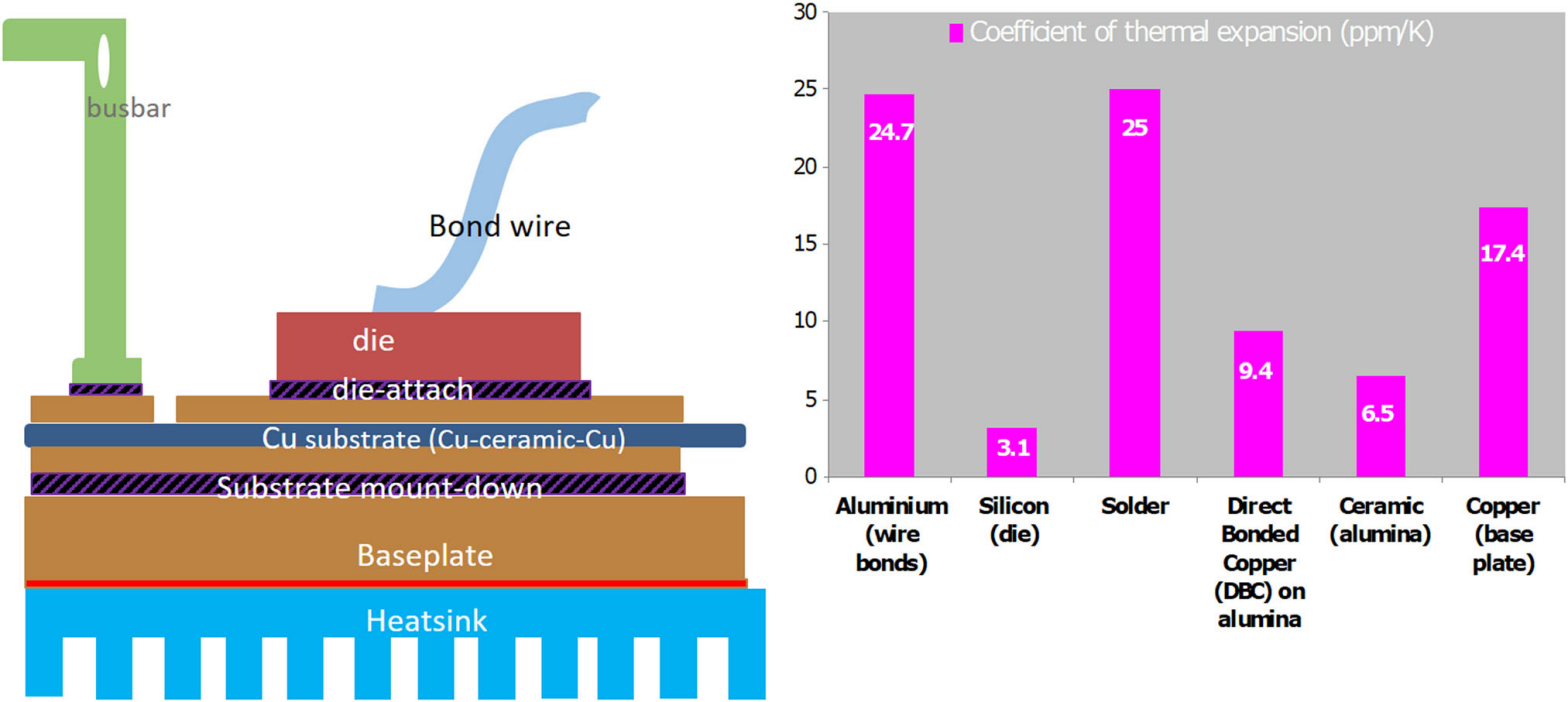
(A) A schematic representation of the constituents of a typical power module and (B) coefficients of thermal expansion of typical power module materials.

Power electronics is an important energy conversion technology which strongly underpins progress within several major industrial sectors including transport and energy generation. These sectors have a central role to play in relation to urgent issues surrounding environmental preservation and global sustainability strategies (Chan, 2006). However, power electronics assemblies are also ostensibly the most critical components in renewables technologies from the point of view of efficiency, performance and cost (H. Wang *et al*., 2012, 2014). A power module, by design, harnesses the intrinsic ability of semiconductors to be ‘switched’ for power conversion and control. However, this fundamental principle also comes with an inherent problem. During operation, heat is generated within the semiconductor due to switching and conduction losses; and although a power module is designed to facilitate the conduction of heat away from active devices and through the package constituents, the semiconductor chip and its surrounding elements invariably undergo cyclic temperature excursions during repeated switching. A further source of strain stems from the wide range of materials which make up a typical power module, and which have vastly differing coefficients of thermal expansion (see Fig. 1). These reliability issues impact primarily the interconnections, such as the attachments which connect the dies to a substrate and form the cathode terminal, and bond wire interconnections (anode terminal) (Ramminger *et al*., 1998; Ciappa, 2002; Otiaba *et al*., 2012).

Presently, soldering is the most widely used die attachment method for power module packaging. However, conventional tin‐based solder alloy die‐attachments are inadequate for the emerging, more challenging (high temperature) wide band gap applications due to their susceptibility to thermomechanical fatigue and creep. Although high lead‐based solders have a higher melting point and are more reliable than tin–silver alloys (Li *et al*., 2014; Tian *et al*., 2014), lead and the chemicals associated with the soldering process (e.g. formic acid) are environmentally damaging, and their removal from existing Restriction of Hazardous Substances (RoHS) exemptions may be imminent (Hunt *et al*., 2014). In these respects, sintered nanosilver die attachments have a number of advantages: (1) superior electrothermal properties and perceived reliability advantages and (2) a more ‘clean’ manufacturing process (Knoerr *et al*., 2010; Hunt *et al*., 2014), requiring lower processing temperatures than solder alternatives (Regalado *et al*., 2019). However, knowledge of their degradation mechanisms and reliability behaviour is only beginning to emerge, and as yet, the authors know of no phenomenological or constitutive models in place to describe their degradation.

Typically, metallurgical cross‐sectioning techniques are the norm for analysis of crack development and microstructural integrity; however, for die attachments, this method is only really appropriate for the through‐thickness plane, as bondlines are often 100 μm or less. Moreover, as only one of an infinite number of potential planes can be examined, there is a risk of misinterpretation of qualitative features, or the exaggeration or underestimation of quantitative information. An additional specific problem in relation to sintered structures is that metallographic preparation can severely compromise the efficacy of porosity and defect quantification significantly through smearing and smudging of pores.

In recent years, X‐ray computed tomography (CT) has revolutionized the nondestructive observation of the internal structure of engineering materials, where increased accessibility and affordability of laboratory based systems with submicron resolution has led to a surge in new perspectives and research output (Zhang *et al*., 2009; Tsuritani *et al*., 2011; Withers & Preuss, 2012; Withers, 2013). The same test sample can be assessed at different stages of its lifetime, thus allowing an accurate representation of microstructural damage evolution under exposure to operational loads to be observed three dimensionally.

Within the context of power electronics reliability, same‐sample experimental methods have a crucial role to play in cradle‐to‐grave fatigue life studies, in deepening our understanding of physical degradation mechanisms, and in so doing providing the necessary data to support the development of models from first principles. Additionally, this approach has the advantage of helping to reveal sources of lifetime variation and quantify their effects and interactions (Arjmand *et al*., 2016a).

Although the use of this X‐ray CT has been prevalent within other scientific fields for a number of decades, its emergence within power electronics packaging reliability studies is relatively recent (Xiao *et al*., 2011; Padilla *et al*., 2012; Agyakwa *et al*., 2014; Li *et al*., 2014; Agyakwa *et al*., 2016; Feng *et al*., 2016; Regalado *et al*., 2019). There are a number of reasons for this. Tomography imaging of a power module package is not easily achieved due to the rather divergent X‐ray absorption characteristics of its low and high Z element constituents, the former including ceramics, polymer flexes and encapsulants, and the latter including copper, silver and tin. Their contrasting attenuation characteristics create nonideal imaging conditions, resulting in a myriad of artefacts which present challenges in relation to subsequent image segmentation and analysis. Furthermore, scan times can be far from optimal, resulting in compromise of the X‐ray counts (intensity) achievable at the detector, or an unfortunate trade‐off between spatial and temporal resolution, potentially hindering the timely perception of early stages of damage. Another challenge usually encountered is the resolution of fine features, such as cracks, voids and discontinuities in relatively large specimens, as large specimens can limit the achievement of optimal geometric magnification. In this work, X‐ray imaging was performed on a Versa‐XRM 500 system, supplied by Carl Zeiss X‐ray Microscopy, Pleasanton, CA, USA, with a maximum electron acceleration of 160 kV. This model is unlike traditional laboratory micro‐CT platforms in that it possesses a turret of objective lenses, and so allows two‐stage magnification (geometric and optical), with large working distances, thus ideally suited for the imaging of power modules and their constituents.

### Prior 3D X‐ray tomography reliability studies of sintered nano‐Ag die attachments

**Figure 2:**
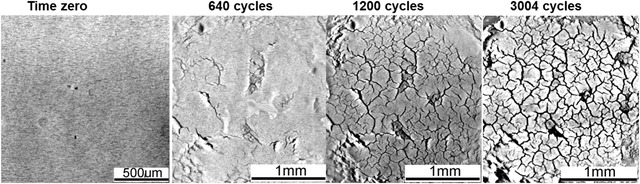
A time series of virtual lateral cross‐sections through a sintered nanosilver die attachment thermally cycled from −55 to 190°C (Li *et al*., 2014).

Even though their role in the enhancement of power conversion technologies is both timely and urgent, the advantageous reliability characteristics of sintered die attachments still require further understanding and modelling. The first temporal 3D X‐ray tomography reliability study under temperature cycling study of sintered nano‐silver (paste‐based) die attachments was carried out by Li *et al*. (2014), which presented previously unseen lateral views of damage evolution which would be unfeasible to achieve using other imaging techniques. These unprecedented perspectives revealed new qualitative characteristics of the damage, including the observation that their formation mechanism and appearance of cracks appeared analogous to those observed in the desiccation of soils (see Fig. 2).

Subsequent X‐ray tomography studies of attachments formed using both sintered film and paste by Y. Wang (2016) made a number of original contributions. Firstly, it was suggested that the transient nature of the sintering process (of the order of seconds/minutes) and the kinetics of densification, a time‐dependent phenomenon, mean that densification of the nanoparticles may not reach completion during the die attachment process. Any subsequent temperature exposure (cycling) then provides the necessary thermodynamic driving force for continued densification, and that cracks and discontinuities which form during thermal cycling are a result of the shrinkage which accompanies this process. Y. Wang also suggested that the crack morphologies and crack paths were associated with both the surface roughness of the substrate, and the *a priori* heterogeneity of the sintered attachment interlayer structure, such as undispersed silver coagulates and other microstructural heterogeneities, as these resulted in nonuniformity of sintering pressure across large areas. Potential reliability outcomes for sintering onto different substrate surface presentations or platings were also investigated (Y. Wang, 2016).

**Figure 3:**
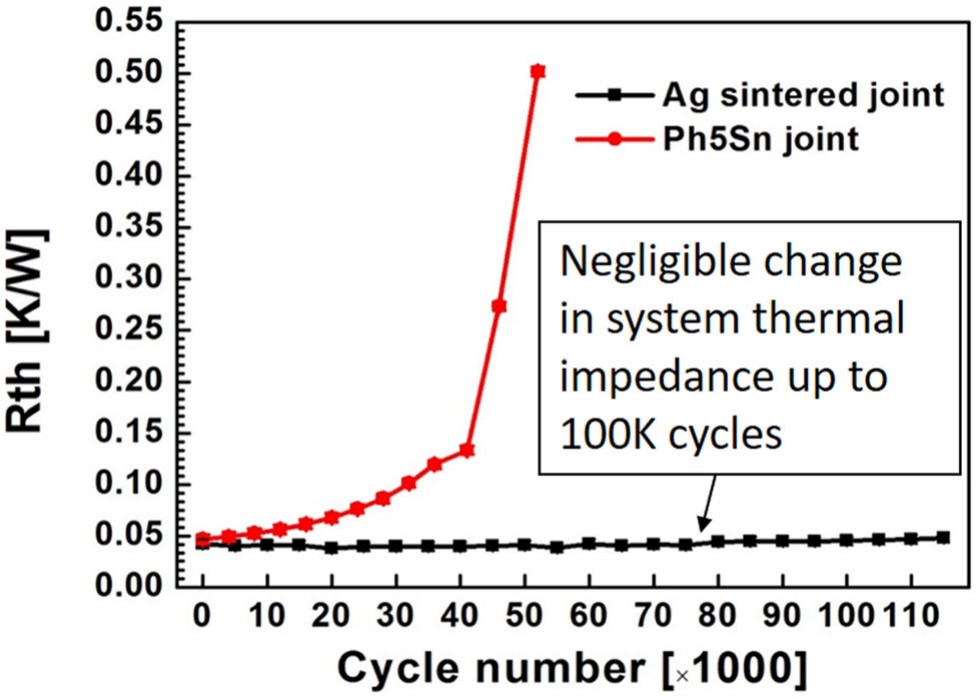
Comparative thermal Impedance behaviour sintered die attach and a high lead solder attachment as a function of number of power cycles (Dai *et al*., 2017).

More recent work in this area was carried out by Dai *et al*. (2018) on a comparative study of solder and nanosilver‐sintered attachments. This study, underpinned by the correlation of 3D X‐ray microscopy data with sequentially evaluated electrothermal characteristics, showed that the effective thermal resistance of a sintered attachment assembly remained relatively unchanged after 100 000 power cycles (Fig. 3). In direct contrast, a high‐Pb solder joint's thermal resistance steadily increased from the outset and yielded a lifetime one tenth that of the sintered attachment.

Dai (2018) also presented reliability data from four sintering trials with a range of sintering times, pressures and temperatures. This range in parameters was intentionally chosen to produce attachments with different levels of bonding strength/porosity, so as to determine the range of reliability outcomes possible, in comparison to solder. Interestingly, their thermal impedance evolution and reliability behaviours under power cycling were remarkably similar. Dai's work provides a strong case for the use of shorter sintering times, lower temperatures and pressures which may be more economically advantageous and more compatible with existing manufacturing lines, as these still produce attachments orders of magnitude stronger than high‐lead solder alloy attachments.

### Aims of paper

This paper represents a continuation of Dai's work and aims to unpack these findings further by investigating the morphological characteristics of the evolving damage by combining 3D damage morphology information with data obtained from other microscopy techniques. Here, we place focus on two specimens in Dai (2018) at opposite ends of the quality spectrum: i.e. (1) a low shear strength, high porosity sample and (2) a high shear strength, low porosity sample. (Details of the processing parameters of these specimens are provided in Table 1.).

**Table 1:** Processing parameters and properties of specimens under study (Dai, 2018)

	Sintering parameters				
Specimen	Temperature (°C)	Pressure (MPa)	Time (s)	Porosity (%)	Shear strength (MPa)
A	300	19.5	9	24.7	52.7
B	220	6	1	50.9	20.5

## Experimental procedures

### Materials

The materials used to manufacture the test coupons used in this study were:

1. CREE CPW4‐1200‐S010B SiC diodes 2.26 mm × 2.26 mm × 0.377 mm in size, with a 1.4 mm Ni/Ag metallization on the cathode (back side) and a ∼4 mm Al metallization on the anode (top side).
2. Aluminium nitride ceramic substrates consisting of a 1‐mm‐thick AlN ceramic tile sandwiched between copper 0.3 mm active brazed copper layers. Additionally, these substrates were finished using a 0.2 μm silver plating to facilitate sintering.
3. The nanosilver film used (Argomax 2020) was 62.3 μm thick with an average particle size of ∼20 nm, and obtained from Alpha Assembly Solutions (Somerset, NJ 08873, USA).

### Sintering procedure

**Figure 4:**
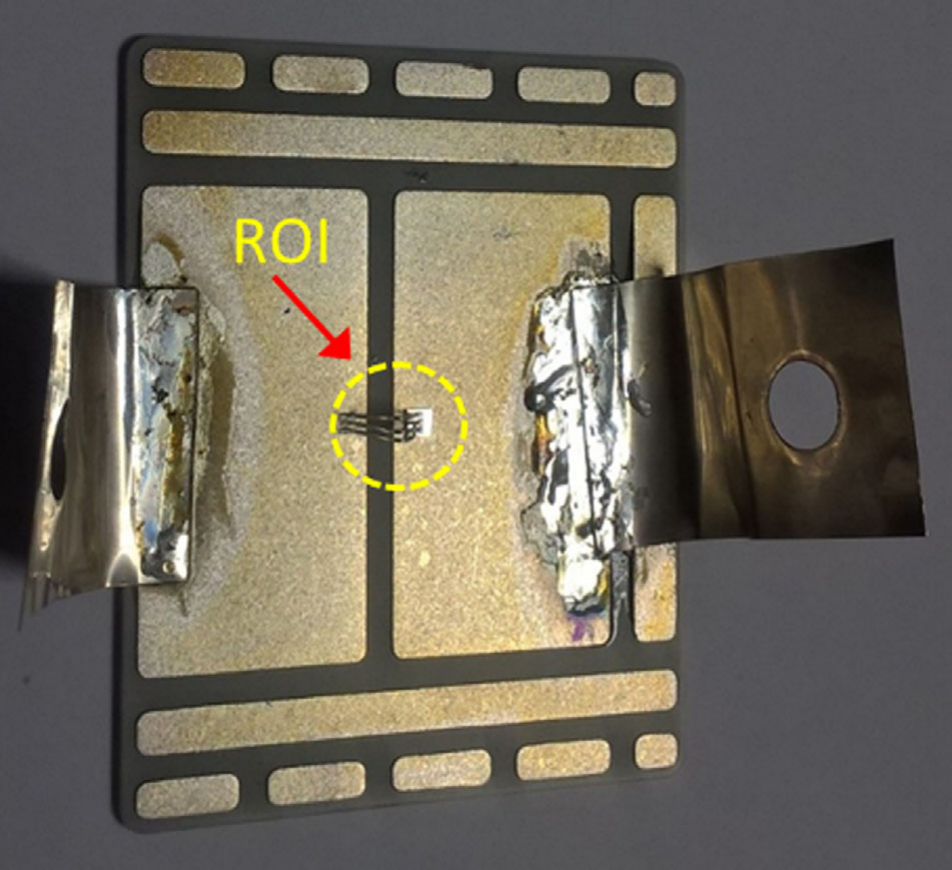
A typical test coupon ready for power cycling and imaging.

The sintering process itself was performed on a Datacon 2200 EVO die bonder. Following sintering, a few aluminium wires were ultrasonically bonded to provide interconnections between the anode of the diode and the appropriate track on the substrate tile. Two strips of 125 μm Ag foil were also soldered on to the substrate to act terminals, providing external circuit connections. Figure 4 shows the finished product. Further details of the sintering process can be found in Dai *et al*. (2017).

### Power cycling and thermal impedance measurement

Power cycling was performed using a Mentor Graphics^®^ Power Tester 1500A, which provides information about the evolution of thermal impedance under cycling (Mentor Graphics^®^ Datasheet [Online], n.d.). A detailed account of both power cycling and thermal characterization protocols can be found in Dai *et al*. (2018). In summary, test coupons were mounted onto a water‐cooled cold plate maintained at 20°C. A 500‐μm polytetrafluoroethylene (PTFE) film was inserted between the substrate tile and the heat sink to artificially increase the substrate‐to‐ambient thermal resistance so as to obtain a large temperature fluctuation, thereby accelerating the evolution of damage. The current was regulated to ensure that the temperature amplitude of 150°C (+50 to +200°C ± 7°C) remained constant. Junction temperature estimations derived from forward voltage drop were utilized to obtain *in‐situ* transient thermal impedance measurements for the as‐sintered device, and then recurrently at different stages of life.

### X‐ray tomography imaging and analysis

**Figure 5:**
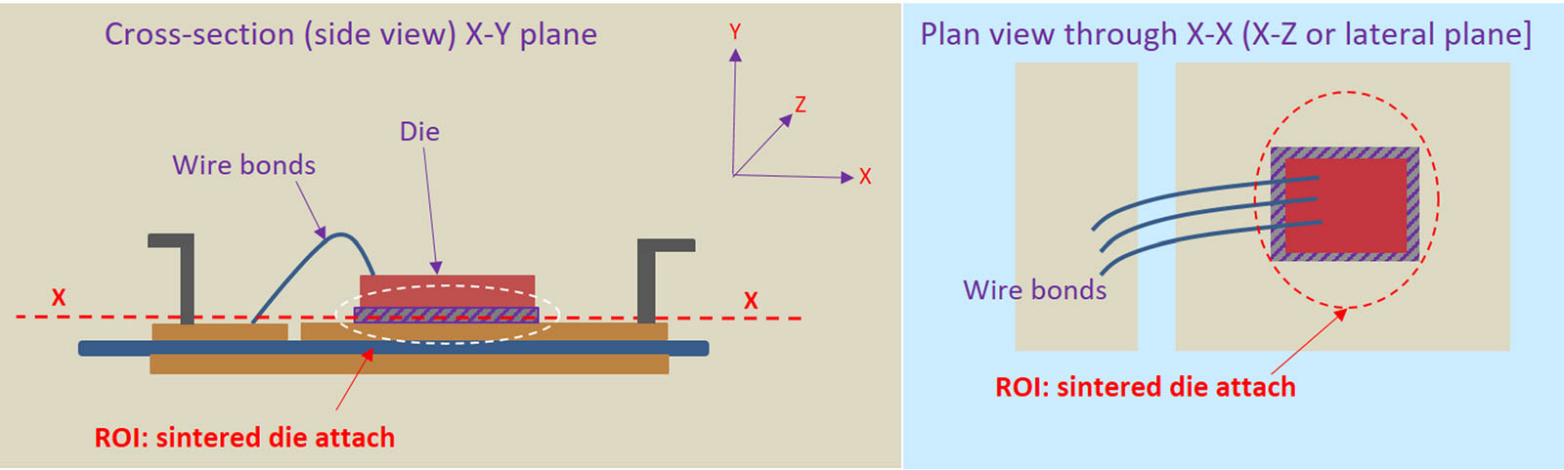
Field of view and region of interest (ROI) in the *X*–*Y* and *X*–*Z* planes.

The development of discontinuities (openings, voids and cracks) within the sintered attachment and at other interfaces during power cycling has been studied nondestructively using 3D X‐ray tomography, using a Zeiss Xradia Versa‐XRM 500 system. The die attachments were imaged prior to power cycling (in the ‘as‐sintered’ condition) in order to provide a basis for comparison. The same test coupons were then subsequently imaged at several stages of thermomechanical fatigue life between 0 and approximately 650K cycles. Imaging was performed using a source voltage of 140 kV and a 4× objective detector. Specimen‐to‐source and specimen‐to‐detector distances varied slightly with each setup but were typically 40 and 70 mm, respectively. An appropriate filter was applied to the X‐ray beam to minimise artefacts. A 2 × 2 camera binning mode was used to capture the images at exposure times per projection ranging between 12 and 22 s. These parameters allowed a spatial resolution of about 2.3 μm to be achieved, which enabled the entire area of the sintered attachment (2.26 × 2.26 mm) to be within the field of view. This spatial resolution was deemed to be adequate for the resolution of emerging cracks and openings. Due to the high aspect ratio of the specimens, full rotation was considered unnecessary; thus, a total of 1601 projections were acquired over a rotation span of 180° for each scan. The region of interest is depicted in the schematic diagram give in Figure 5.

The 1601 projection images were reconstructed using *Xradia‐Zeiss 3D Reconstructor* software which uses a filtered backprojection algorithm. The reconstruction procedure included determining the centre shift (the offset in pixels of the rotational axis from the centre column of the detector), and occasionally applying a correction for beam hardening.

The tomography datasets were visualized and multiple virtual cross‐sections were obtained using *Xradia‐Zeiss 3DViewer*. Further visualization, segmentation, surface generation and semi‐quantitative area and volume analyses were carried out using *Avizo Fire 9.0.1* software. The segmentation procedure involved extracting a subvolume defining the region of interest, creating a mask, applying a nonlocal means filter and utilizing a range of thresholding tools to select and isolate features of interest, which were then rendered to aid 3D visualization.

### Metallurgical cross‐sections for optical and scanning electron

To obtain correlation of the virtual *X*–*Y* plane perspectives with other microscopy techniques, *Specimen B* (see Table 1) was metallurgically sectioned after approximately 650K power cycles and after obtaining nine sequential tomography datasets. The sample was then mounted in edge‐retentive epoxy resin and cured at room temperature for 24 h. It was then slowly and carefully mechanically polished on a Buehler Metaserv automatic polisher. The specimen was grounded successively with 1200, 2500 and 4000 grit silicon carbide papers, and 3 and 1 μm diamond slurries, ensuring that damage from successive grinding/polishing steps was completely removed before progressing. A final polish was achieved using a 0.06 μm colloidal silica suspension using a Buehler Vibromet‐2 vibratory polisher. To reveal porosity and cracking, the sample was examined using optical and scanning electron microscopy (SEM) in its as‐polished state; the relationship of cracks and pores to the grain morphology of the copper substrate was then examined by swab‐etching with alcoholic ferric chloride solution. Optical microscopy of the metallurgically cross‐sectioned specimen was carried out using a Nikon Optiphot metallurgical microscope, and SEM imaging was performed using a Hitachi TM3000 desktop Scanning Electronic Microscope (SEM, Hitachi HighTech Minatoku, Tokyo, Japan).

## Results and discussion

### Damage formation within the sintered attachment layer

**Figure 6:**
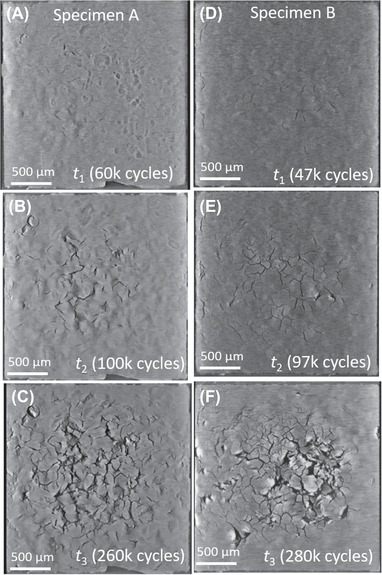
Overview of tomography datasets of *Specimens A&B*, showing virtual cross‐sections from the *X*–*Z* (lateral) plane.

Time series of lateral and through‐thickness virtual cross‐sections give an overview of the chronology of crack development under thermal cycling within the two specimens under investigation (see Fig. 6). Early stages of damage are characterized by smaller crack apertures and therefore poorer contrast relative to the background. As damage progresses and cracks and voids widen and deepen, the greyscale values increase accordingly. X‐ray tomography is particularly suited to the study of these cracks because of the high absorption contrast between the crack openings or ‘separating surfaces’ (air) and silver, which improves as the cracks widen. The intensity and range of the greyscale values also relate to local variations in material density. The ‘texture’ of the images visible in Figure 6 (differing levels of X‐ray absorption) in this case is thought to be related to nonuniform packing density due to depressions on the substrate, as detailed below.

**Figure 7:**
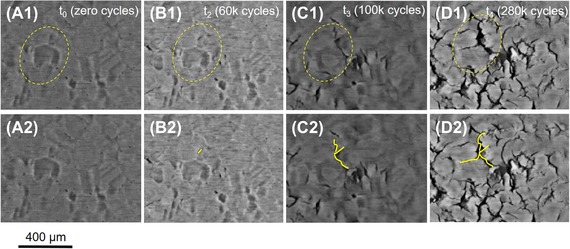
Magnified virtual cross‐sections showing relationship between crack initiation within the sintered attachment and the underlying copper grain structure within *Specimen A*. (Figs. 7A2–D2 are a duplication of Figs. 7A1–D1, for clarity.)

**Figure 8:**
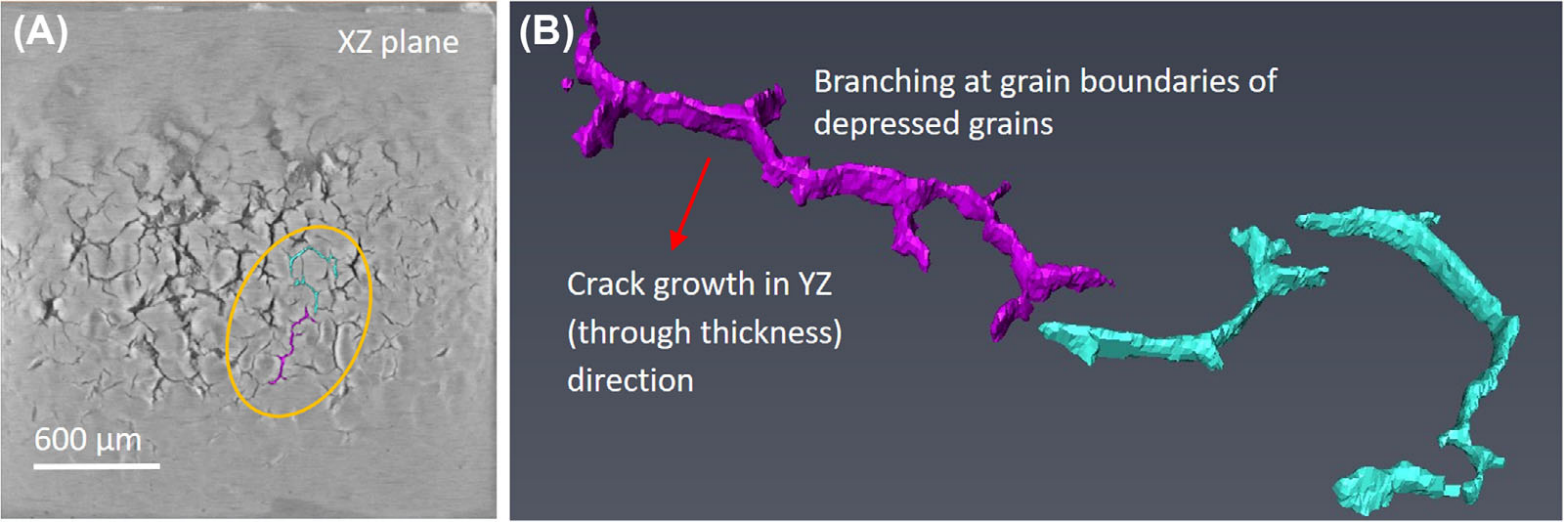
Segmented crack networks depicting crack branching at the grain boundaries of depressed grains in *Specimen A*.

The primary difference in the structures of the two specimens is the appearance of this texture within the lateral cross‐sections. To explain, *Specimen A*, bonded using high pressure, high temperature/long sintering time, shows a pronounced imprint of the roughness originating from the grain structure of the copper substrate tile beneath, that is, the relative depths of the different grains. Such imprinting is not obvious in *Specimen B*, the ‘lower quality’ specimen (lower sintering temperature/pressure and shorter period). It can also be seen that the least dense areas (darker pixels) are associated with crack initiation. These observations are further substantiation of observations first made by Y. Wang (2016) on sinter‐attached large area (13 × 13 mm) dies. In *Specimen A*, these less dense areas appear related to the copper grain boundaries, triple points in particular. Cracks also coincide with grain boundaries, and their propagation also appears to occur preferentially along the grain boundaries at this depth. This is exemplified in the magnified series of images shown in Figure 7 which chronicle the damage evolution in *Specimen A*. Figures 7(A2)–(D2) are a replication of Figures 7(A1)–(D1), to allow the annotated images to be viewed simultaneously with the unmarked images. In the *a priori* virtual slice (A1), a feature is circled which consists of dark pixels (low density) region in the shape of a depressed grain, and its interconnecting boundaries. The beginnings of a network of cracks can be seen in Figure 7(B2), corresponding to the darkest pixels within the feature, along a grain boundary. In Figures 7(C2) and (D2), and after further power cycles, further cracks appear and form branches at the other grain boundaries of the triple point identified in Figure 7(A1). To illustrate this further, segmentation of another network of cracks in *Specimen A* after 260K cycles clearly depicts the branching of cracks at the boundaries of subsurface copper grains (see Fig. 8).

The evolution of damage in *Specimen B* is also characterized by numerous Y‐shaped cracks (Figs. 6E and F); however, in the absence of obvious grain structure imprint, it is uncertain whether the Y‐shaped cracks are due to an underlying grain structure/correlate with triple grain boundaries as in *Specimen A*. A key difference between the two specimens, however, is that even with similar amounts of preexisting damage, cracks are significantly wider within *Specimen B* after similar numbers of cycles, along with increased densification in the vicinity of said cracks.

**Figure 9:**
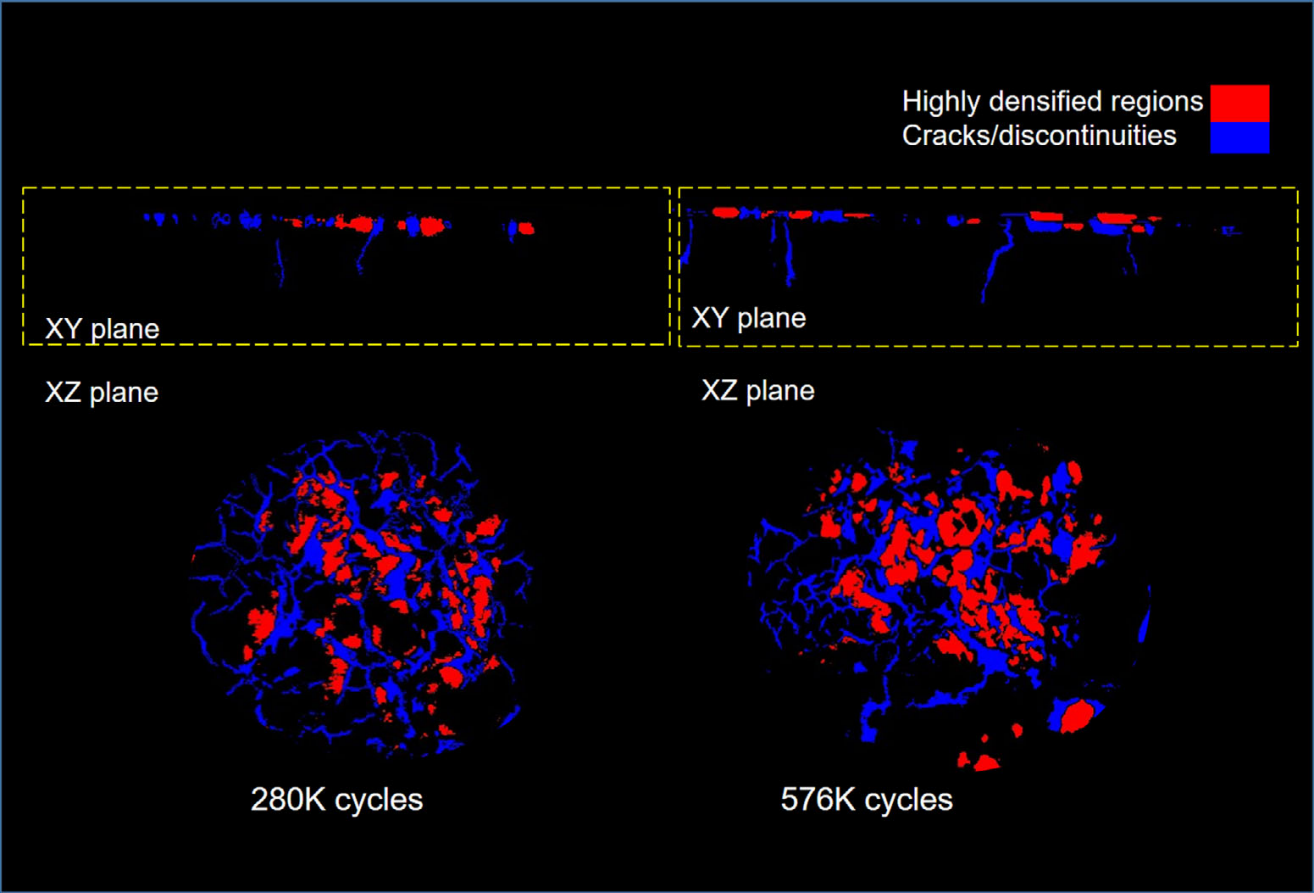
Segmented tomography datasets from *Specimen B* showing the highest pixel intensities in close proximity to widest/most developed cracks.

**Figure 10:**
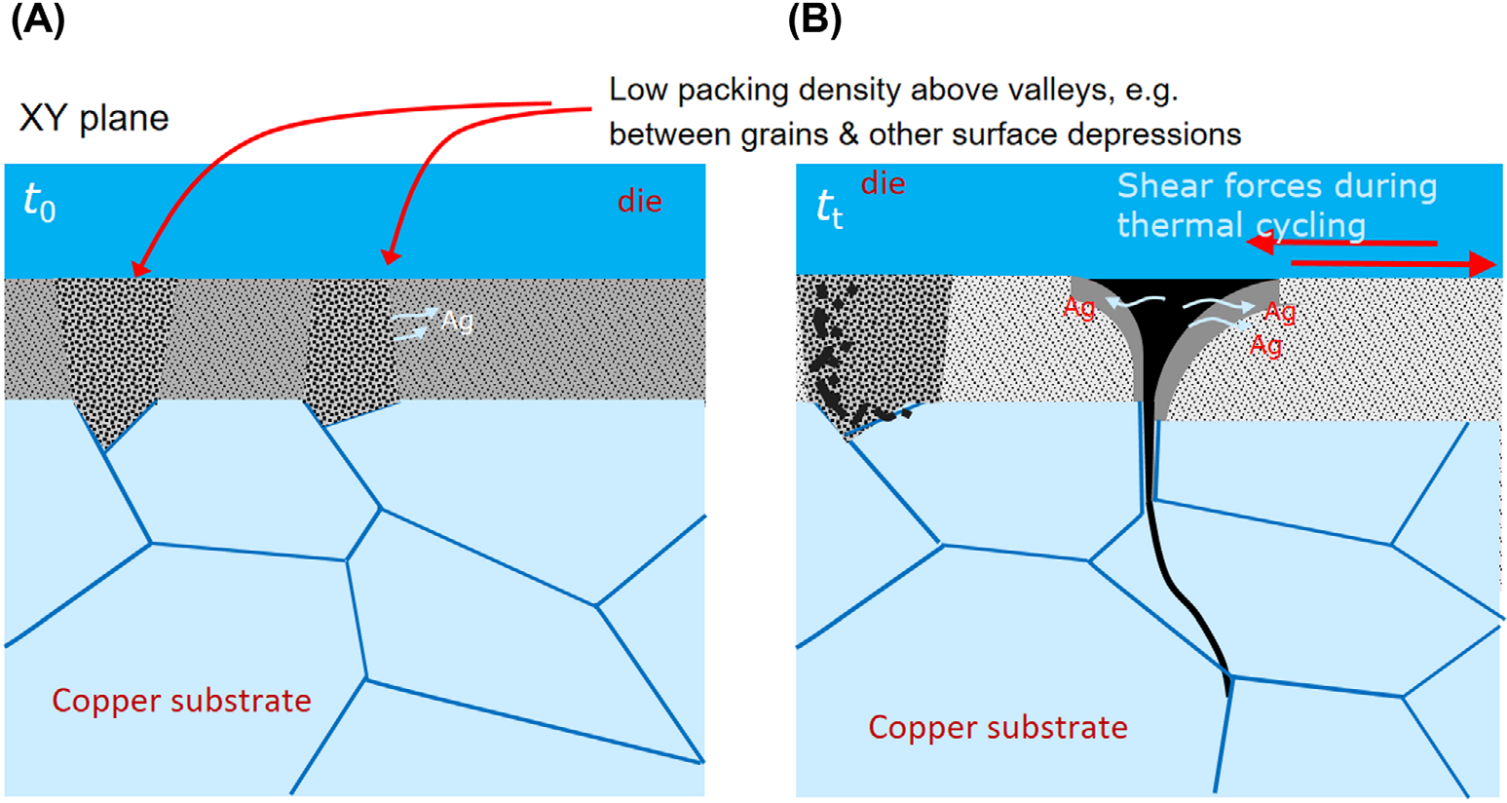
Schematic representation of the proposed mechanism for densification, crack initiation and propagation.

As observable in Figures 6(C) and (F), in both samples, the highest pixel intensities occur directly adjacent to the widest and most developed cracks for both specimens, though with greater prominence in *Specimen B*. In Figure 9, two segmented tomography datasets from *Specimen B* highlight this, and clearly show highest pixel intensities in close proximity to most developed cracks. This, evidently, signifies the ongoing densification during power (thermal) cycling, as previous work has suggested (Y. Wang, 2016), and the activation energy which is relatively low for nanoscaled particles (Dai, 2018). The lower pressure and temperature of sintering imply less energy and time for densification, and that surface diffusion, a nondensifying mechanism, may play some role in the coalescence of the nanoparticles (Bordia *et al*., 2017). Thus, the attachment microstructure, which remains in an intermediate, thermodynamically unstable stage of incomplete sintering immediately postmanufacture, continues to densify under operation. The increased crack aperture which accompanies both crack growth and densification may be associated with the diffusion of silver atoms away from areas of low silver concentration (low density, dark pixel areas) to high‐density regions, a thermodynamically driven process resulting in the reduction of interfacial or surface energy. A schematic representation in Figure 10 illustrates this proposed mechanism. By this model, crack widening may continue beyond the point of the stabilization of their growth. Ostensibly, the analogy with drying soil (e.g. a cracked dry riverbed) also applies in the context of their kinetics. For example, Lu *et al*.’s (2016) study of clay under freeze‐thaw cycles showed that the development of new, interconnected polygonal networks of cracks reached a point of stabilization, after which the main damage phenomenon observable was their widening.

### Interaction of cracks at interfacial boundary and propagation through substrate

**Figure 11:**
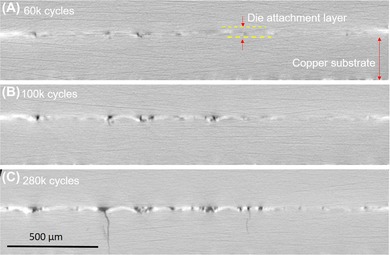
Virtual cross‐sections showing of damage evolution from the same *X*–*Y* (through‐thickness) plane within *Specimen A*.

Figure 11 shows a sequential set of virtual cross‐sections of the *X*–*Y* or through thickness plane for *Specimen A*, looking at the same virtual slice after different stages of life with increasing damage. The bondline, which is about 30 μm thick, can be seen, containing dark pixels, which correspond to cracks. Brighter pixels adjacent to cracks are also evident in this perspective, and these increase in brightness as the cracks grow and deepen, mirroring observations made in the lateral cross‐sections in Figures 6 and 9. Secondly, cracks originate from within the sintered die attachment layer, but begin to intersect and traverse the interface between the attachment and the copper substrate around 100K or so cycles. This may be plausible driven by the reversal between compressive and tensile stress states within the copper layer as it begins to crack. A similar observation was made by Regalado *et al*. (2019), who noted the formation and propagation of copper cracks formed directly beneath the regions damage accumulation within a sintered die attachment subjected to cycling on the solder.

**Figure 12:**
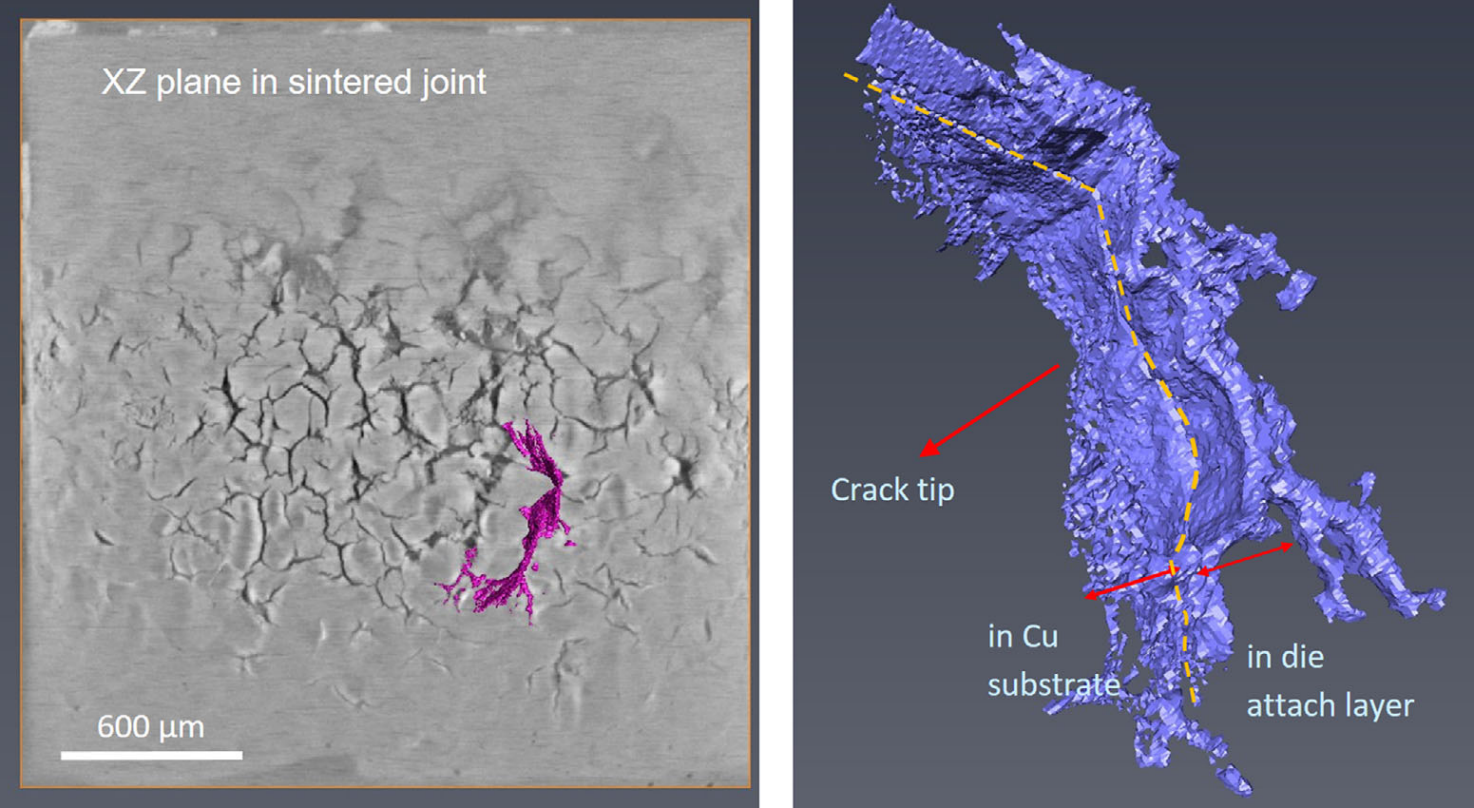
3D rendering of subsampled crack front from *Specimen A*, showing crack morphology.

A small crack network of cracks in *Specimen A* has been segmented out to provide a different perspective (Fig. 12). As its 3D surface shows, cracks appear as thin, vertically orientated sheets in the through thickness direction. This one, like several others after 260K cycles, extends several hundred microns into the copper layer, as can be recalled from Figure 11.

**Figure 13:**
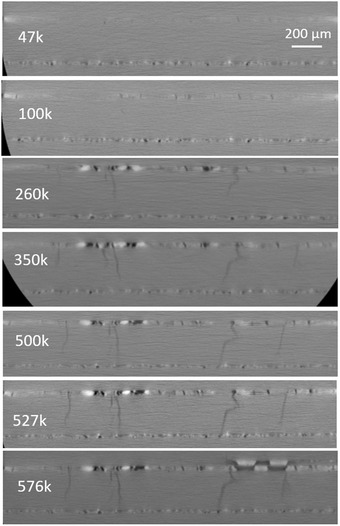
Virtual cross‐sections showing of damage evolution from the same *X*–*Y* (through‐thickness) plane within *Specimen B*.

Like Figure 11, Figure 13 is a chronological series of virtual cross‐sections of the *X*–*Y* or through thickness plane within *Specimens A*. These also show a sintered bondline around 30 μm thick. Again, cracks are initiated within the sintered attachment layer, but eventually cross the interface into the copper layer, and rapidly propagate through. In comparison with the sintered layer, crack propagation through the copper layer appears more rapid. In fact, common to both *Specimens A* and *B*, a number of cracks had traversed the entire thickness of the copper layer (up to 300 μm of growth) within 150K cycles. Certain cracks in the copper appear almost vertical are unattenuated in their movement, whereas others are more tortuous.

**Figure 14:**
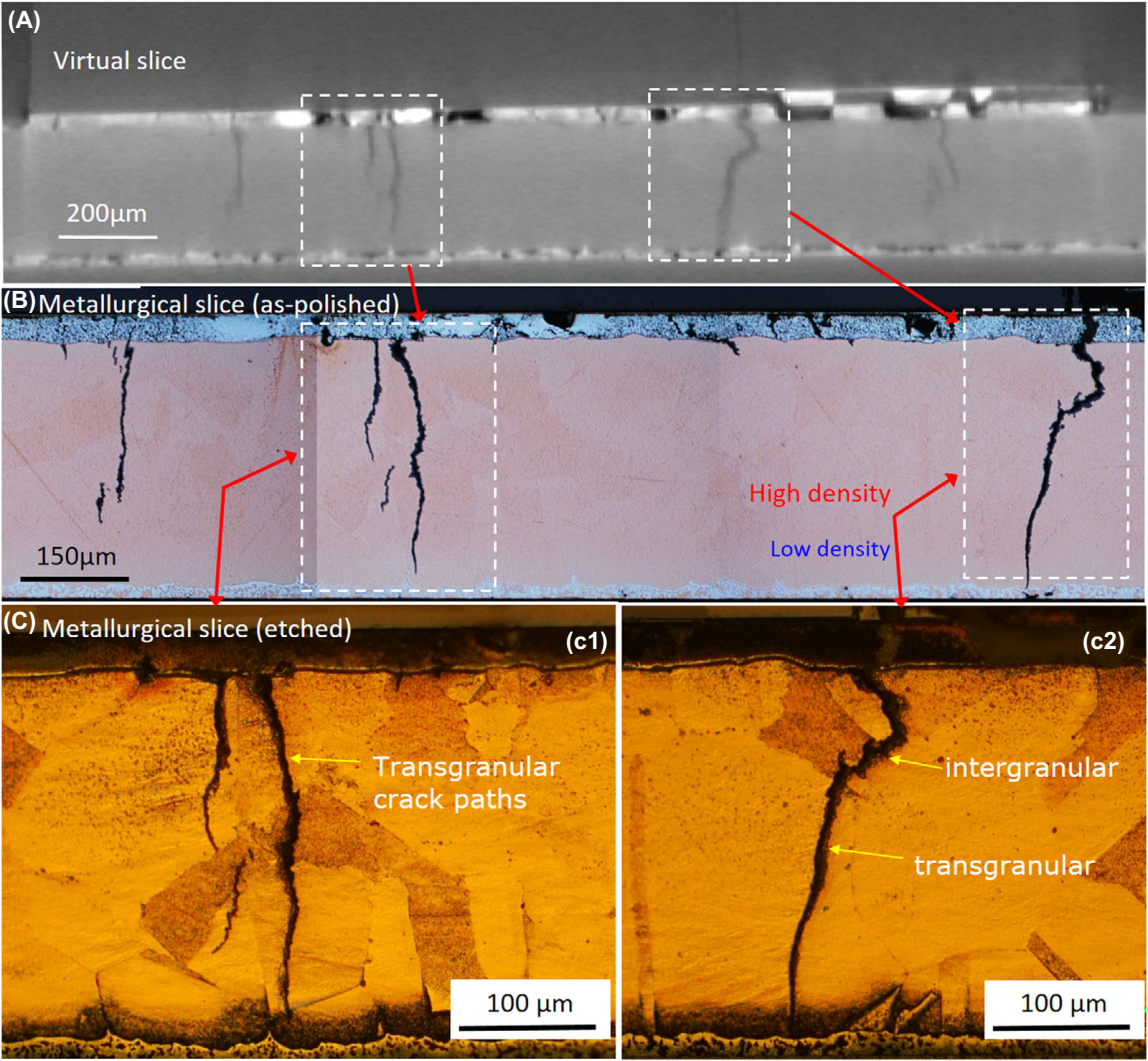
As‐polished and etched optical micrographs of *Specimen B* after 650K cycles, showing cracks within the sintered and copper layers.

**Figure 15:**
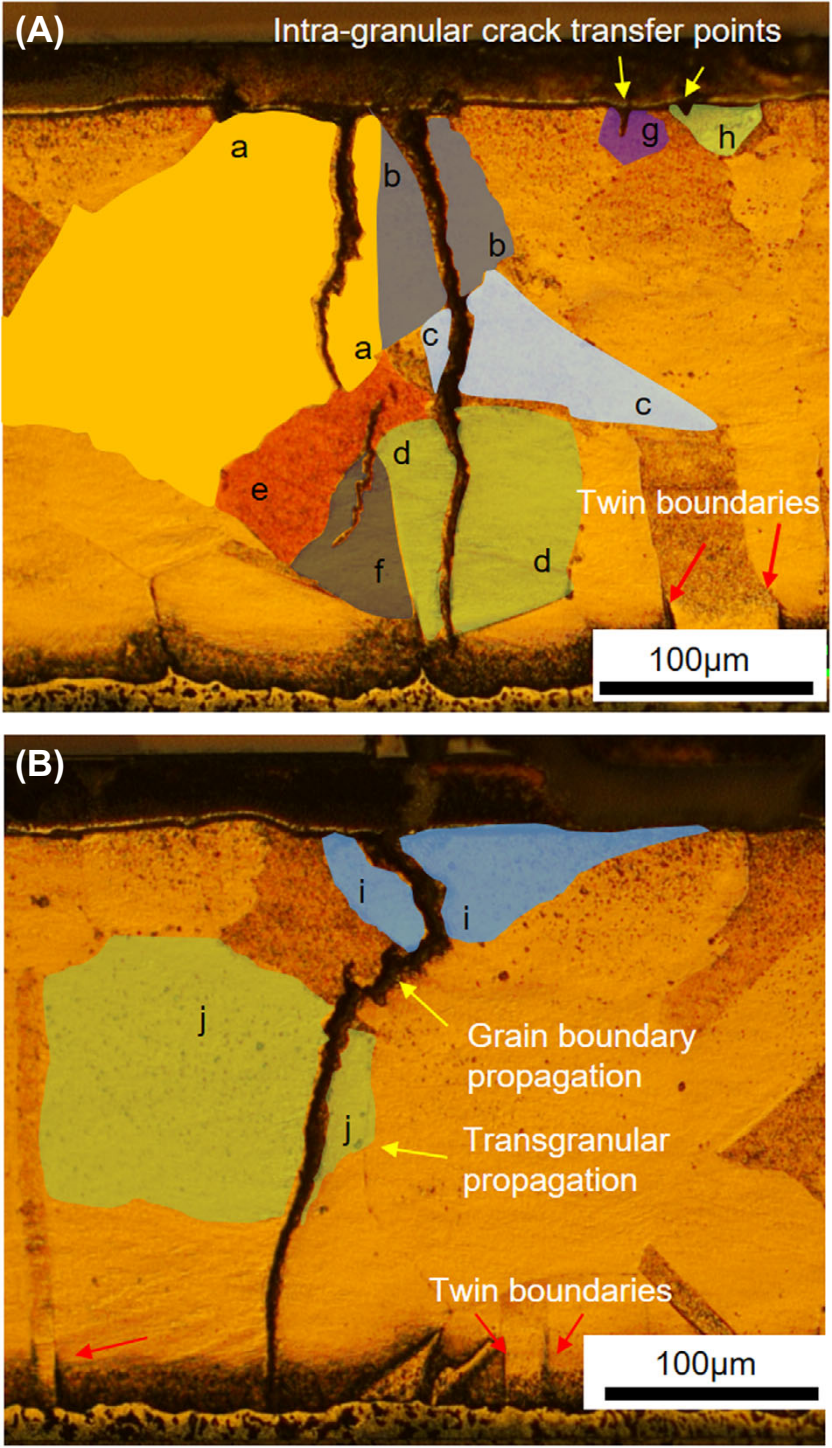
Visualization of relationship between crack propagation paths through the copper substrate of *Specimen B* after 650K cycles.

To further understand the mechanisms governing the evolution of the observed thermomechanical fatigue damage, correlative microscopy was performed using *Specimen B* following the termination of power cycling at approximately 650K cycles. As‐polished and etched metallurgical cross‐sections in the *X*–*Y* (through‐thickness) plane are presented in Figure 14, the exact same plane presented in the virtually in Figure 13. The relationship between the advancing cracks and the grain structure of the copper can be examined in Figure 14(C). The copper microstructure is characterized by large grains approximately 50–150 μm in size, as expected for actively brazed copper subjected to prolonged temperature cycles (Arjmand *et al*., 2016b), and features deformation twins. To illustrate the propagation paths more clearly, it is helpful to look at another representation, as shown in Figure 15, of the micrographs in Figure 14(C) in which grains are annotated and colour‐coded, so that cells belonging to the same grain are more easily identifiable. The group of cracks (C1) in Figure 14(C) (i.e. Fig. 15A) appear decisively and entirely transgranular. However, the propagation mode is less clear cut for the subselection of cracks C2 in Figure 14(C) (i.e. Fig. 15B). In the latter, the crack path through the copper appears to change direction, perhaps transitioning between intergranular and transgranular modes. The varied characteristics of crack movement through the substrate suggest heterogeneous states in terms of the plastic strain of the grains (Robertson & Tetelman, 1960). Transgranular crack paths can be typical of cyclic loading (Turnbull & de los Rios, 1995; Tromans & Sun, 1996), and are brought about by competing mechanisms of ductile crack nucleation, growth by slip deformation mechanism, and brittle cracking by cleavage (Becker & Lampman, 2002). For both crack propagation modes, there are a number of parameters at play, and its occurrence is dependent on orientation with respect to applied stress and response to plastic strain (stacking fault energy, grain size and interstitial/trace solute atoms) and the interaction of the crack with a typically inhomogeneous microstructure of grain boundaries, secondary phases, defects and so on. Deformation twins, which are present in the copper substrate, can restrict the amount of plastic deformation and can also be a source of cleavage cracks (Becker & Lampman, 2002). The propagation of the crack front through grain ‘*a’* in Figure 15 appears to halt on coming into contact with a grain boundary. Grains ‘*b*’, ‘*c*’, ‘*d*’, ‘*i*’ and ‘*j*’ in Figure 15 are traversed and completely separated by the same crack front, which only terminates once it encounters the ceramic layer. Grains ‘*e*’ and ‘*f*’ are partially traversed by a crack front.

**Figure 16:**
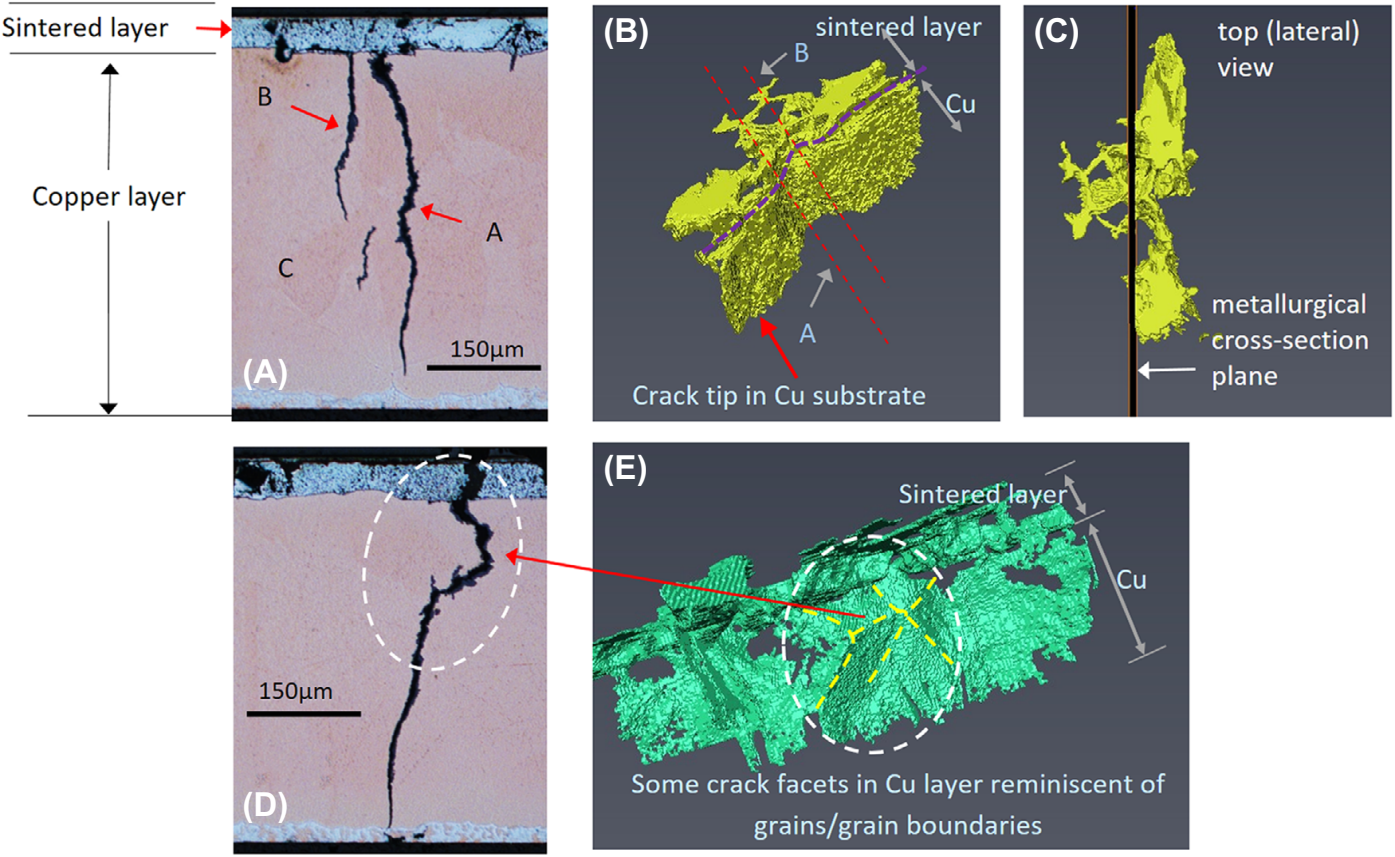
Correlative visualization of cracks within *Specimen B* after 650K cycles. (A) and (D) are metallographic cross‐sections correlating with 3D renderings of the cracks (B), (C) and (D).

The two crack groups in Figure 15 have been segmented out from the bulk and are presented in Figure 16 to examine their morphologies further. The 3D forms or shapes of the cracks observed are recognisably similar to the 2D perspectives. However, from the 3D rendering, it becomes apparent that seemingly separate and unconnected cracks (A, B and C in Fig. 16A) when viewed in two dimensions are, in fact, part of the same crack front (Fig. 16B), emphasizing the importance of 3D perspectives (Ludwig *et al*., 2003; Phung & Spear, 2019). Figure 16(C) is a top down (lateral view) and shows the metallographic sectioning plane.

**Figure 17:**
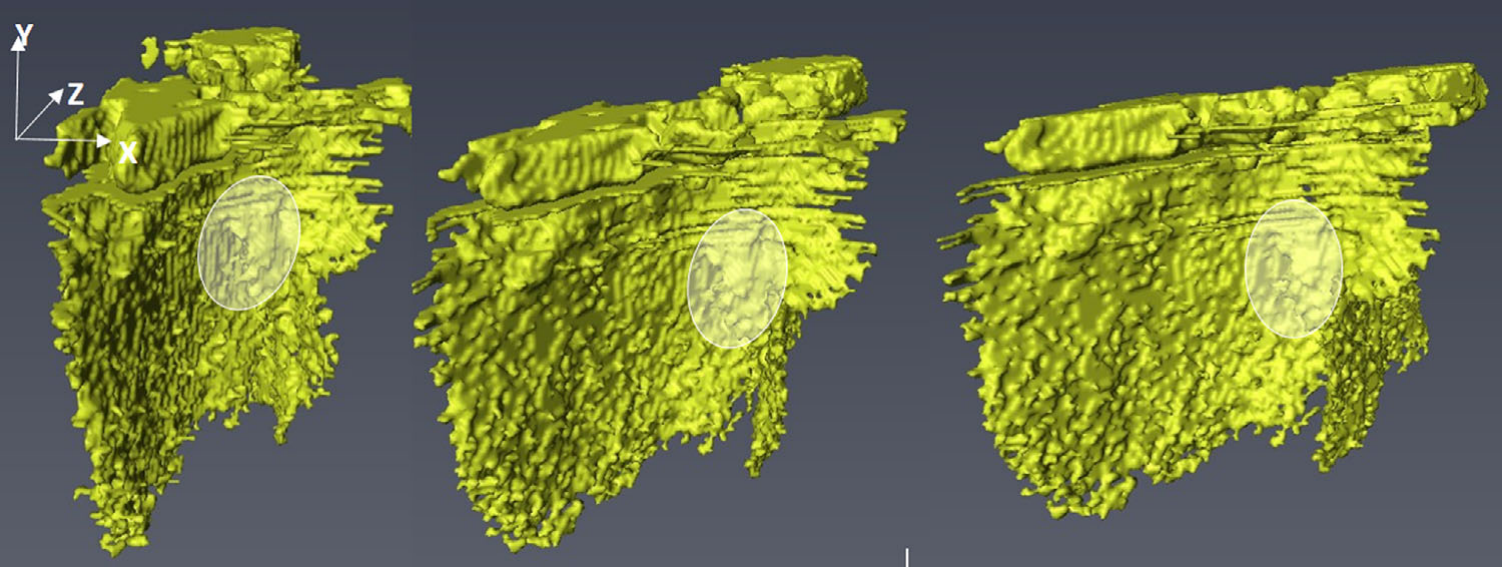
Clockwise rotations of 3D renderings highlighting the crack in grain of Figure 15(A).

In Figure 16(D), some crack faces within the copper substrate appear suggestive of the facets of 3D grains, and correlate with regions in which the crack path was intergranular. But as propagation mode through the copper appears predominantly transgranular, the multiple and differently orientated crack surfaces may also be due to the different orientations of cleavage planes within the copper grains (Becker & Lampman, 2002; King *et al*., 2011), thus resulting in the partial tortuosity of the crack. The energy required for a crack to traverse a grain boundary is influenced by the misorientation between the two grains. Such halts or deflections are said to occur frequently along the crack front in the case of small cracks (Ravichandran & Li, 2000). Examination of different clockwise rotations of the 3D‐rendered crack surface in Figures 16(B) and (C) confirms, as shown in Figure 17. Here, the crack front in question (and other cracks) shows deflections at different points. All the cracks retain their high aspect ratio and orientation with respect to the bondline as they travel through the copper layer.

**Figure 18:**
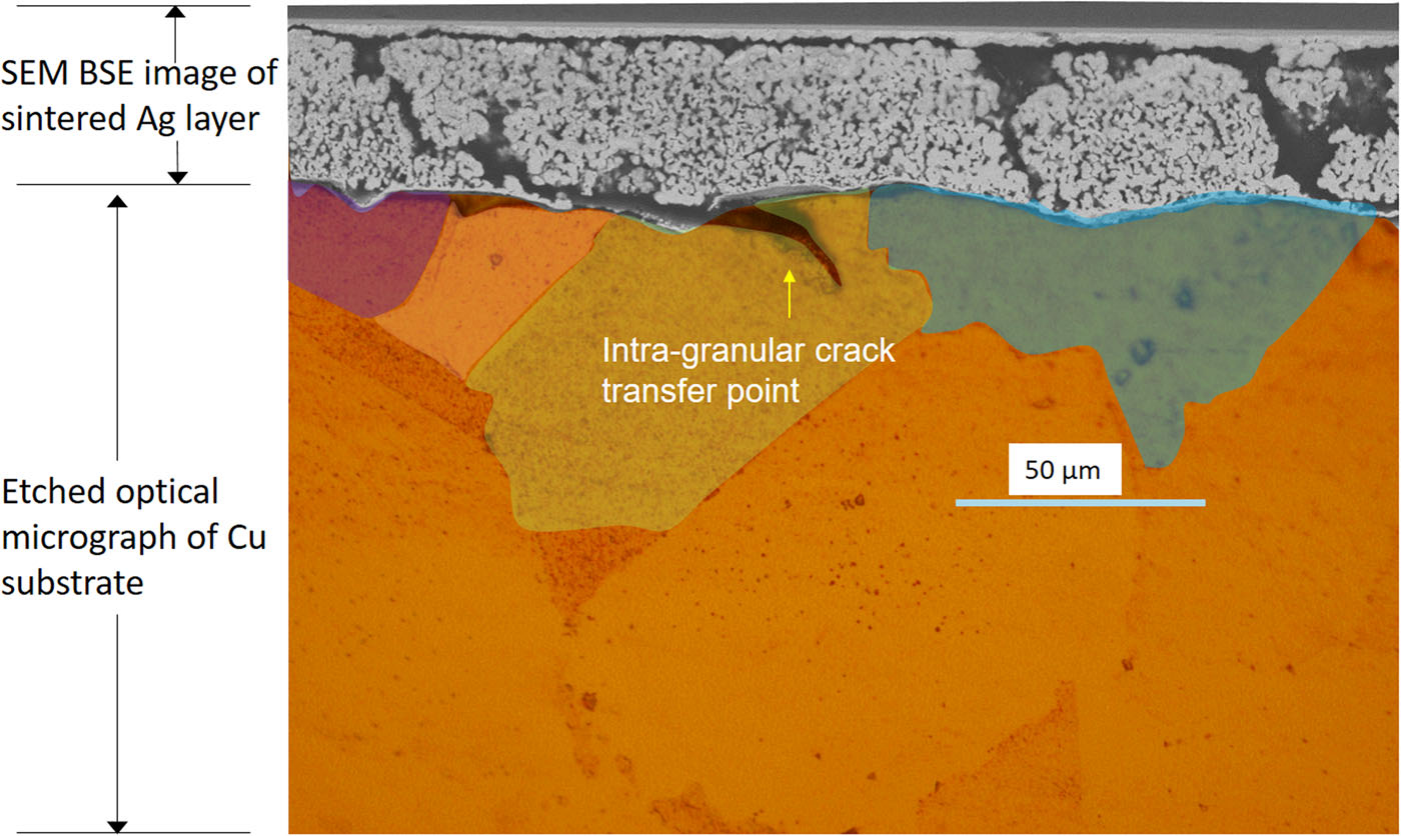
Multimodal overlay of the transition of cracks between the sintered silver attachment and copper substrate layers.

To clearly see the interaction between the origin of the cracks within the sintered attachment and their intersection with and subsequent crack propagation through the copper, it is helpful to merge an SEM image of the sintered layer with a corresponding etched optical micrograph of the substrate (the same slice), as shown in Figure 18. This is because the ferric chloride etch severely erodes the sintered Ag layer and prevents optimal simultaneous views of both the sintered layer and the substrate. An additional advantage is providing information further up the resolution scale. From this integrated image, it can be observed that there are valleys or depressions on the substrate surface, many *intra‐granular* (i.e. not on grain boundaries), which match up with low‐density regions within the sintered attachment (where near vertical cracks form) and which become the points for the transfer of the cracks into the substrate. Similar intragranular crack transfer points were visible in Figure 15, in grains ‘*g*’ and ‘*h*’. Furthermore, lateral cracks which develop at both interfaces of sintered attachment but are not easily resolved in the *X*–*Y* plane virtual slices are clearly visible here. For *Specimen B*, it is possible that surface roughness with origins other than the copper substrate, such as scratches and other superficial imperfections, may play a role. However, this suggestion requires further investigation.

## Significance of findings and conclusions

There are a number of key observations from this work. Two specimens with contrasting bulk porosities and shear strengths were analysed. Microstructural evidence for continued densification during thermomechanical exposure and its relationship with crack development has been presented. The driving force for this phenomenon appeared more prominent in the specimen sintered using a lower temperature/pressure and shorter time, and which was more porous to start with. The surface roughness of the copper substrate leaves a strong imprint in the sintered attachment for the specimen bonded with higher pressure/temperature. 3D renderings of the cracks highlight their high aspect ratio and near‐vertical orientation in the through thickness direction. Cracks initiate within the sintered attachment layers but eventually transfer into the copper substrate primarily at intragranular locations which may be have been *a priori* surface imperfections. Both transgranular and intergranular propagation modes were observed within the copper layer.

In conclusion, the morphology and defining features of sintered nanosilver die attachments are pointers both to their processing conditions and to how they subsequently fare under service. Connecting microstructural data on different points of the resolution spectrum and within a temporal framework elucidates the structure–property relationship and offers new explanations the reliability behaviour of sintered attachments. It is clear from this analysis that sintered die attachments require a different treatment from solder alloy attachments in terms of modelling their reliability, as their damage mechanisms are markedly different. This preliminary study sets the stage for the development of strategies to quantify and model the relevant degradation mechanisms. Future work will harness electron backscatter diffraction (EBSD) and/or diffraction contrast tomography as tools to complement 3D tomography data, and enable more information about the crystallographic aspects of damage evolution and fracture mechanics to be obtained.
